# Does dynamic navigation system preserve more dentine? – A systematic review

**DOI:** 10.1186/s12903-024-04450-z

**Published:** 2024-06-10

**Authors:** Akshayraj Langaliya, Selvakumar Kritika, Aarshvi Shah, Jinali Shah, Sekar Mahalaxmi

**Affiliations:** 1 Department of Conservative Dentistry and Endodontics, AMC Dental College and Hospital, Ahmedabad, Gujarat India; 2 Department of Conservative Dentistry and Endodontics, SRM Dental College, Bharathi Salai, Chennai, India

**Keywords:** Accuracy, Access cavity preparation, Dynamic navigation system, Dentin preservation, Free hand, Static guided endodontics

## Abstract

**Objective:**

This systematic review aims to comparatively analyse the amount of dentin removal by free hand and static guided endodontics with dynamic navigation system (DNS) in endodontic access cavity preparation.

**Methods:**

The systematic review was conducted according to Preferred Reporting Items for Systematic Reviews and Meta-Analyses (PRISMA) 2020 guidelines. Based on the structured PICO framework of “Comparative evaluation of dynamic navigation system (I) to freehand (C) and static guided endodontics (C) in endodontic access cavity preparation on the preservation of tooth structure (O) when assessed on permanent human teeth (P)”, the keywords were formulated and the articles were retrieved from three databases namely PubMed, Scopus and Embase, based on the keywords from the time of inception of DNS till June 2023. The risk of bias assessment was done using a modified Joanne Briggs Institute checklist, which evaluated domains such as randomisation, sample size, image acquisition using CBCT, angulation, accuracy and time taken. As the data was heterogenous, a quantitative meta-analysis was not performed.

**Results:**

Initially, 174 articles were retrieved from the three databases, 30 duplicates were removed, after title check 108 articles were excluded and following abstract check only 10 articles qualified for full text analysis. On reviewing the 10 full text articles, 5 articles were excluded and the remaining 5 articles were subjected to the risk of bias analysis which showed that 2 articles displayed low risk of bias and three articles showed high risk of bias. The RoB analysis revealed that only 2 studies evaluated the preservation of dentin in terms of accuracy, angulation and time taken proving the increased precision with minimal loss of tooth structure using DNS. In both the studies, DNS proved to be superior to free hand technique in terms of precision, accuracy and efficiency in locating the canals during access cavity preparation with maximal preservation of tooth structure.

**Conclusion:**

With the minimal literature evidences, the present systematic review highlights maximal preservation of dentin using DNS. However, further *invitro* and *invivo* studies comparing the free hand, static guided endodontics to DNS must be carried out for its translation into clinical practice. Clinical Significance: Dynamic navigation system provides maximal preservation of dentin during access cavity preparation.

## Introduction

Integrated approach to medical treatment with the use of technology is the future of medicine; with the same applying to dentistry as well. Guided treatment approach was first introduced in implantology, which has influenced its use in endodontics as well, and has paved way for accurate treatment planning and execution, where computed tomography is used to replicate the tooth and surrounding structures via a radiographic template [1]. Guidance in dentistry is basically of two types: Static and Dynamic. In the conventional static guided system, a fixed surgical stent supplemented with Computer-Aided Design/ Computer-Aided Manufacture (CAD/CAM) is clinically employed. However, its inherent disadvantage is that once planned, the process cannot be altered [2]. Static guided access cavity preparation results in 60% peripheral/ tangential deflection due to the inability to alter the predetermined position of the drill [3]. On the other hand, Dynamic Navigation System (DNS) advocates the use of a computer-aided surgical navigation technology with stereoscopic tracking camera which guides the operator’s instrument to retain the ideal position and angulation, while reaching the required depth [4–6]. The system integrates surgical instrumentation and radiologic images with the help of an optical positioning device managed by a computerized program enabling the operator to target position according to the pre-treatment trace obtained from the preoperative planning software and to maintain the predetermined treatment plan during its execution [7, 8].

In endodontics, DNS is used in locating calcified canals, conservative and precise access opening with minimal invasive endodontics, surgical endodontics that includes root-end resection surgeries, bone trephination for apicoectomy surgeries, for delivery of local anaesthesia, etc [9, 10]. Connert et al. proved that in conventional access cavity preparation, there is five times higher substance loss when compared to guided endodontic access cavity preparation. This could be due to the excessive removal of tooth structure leading to decreased fracture resistance and deformability [11]. The amount of tooth structure removed is directly correlated to the tooth biomechanics against the occlusal forces.

Literature reveals several systematic reviews, meta-analysis and scoping reviews which have emphasized the overall use of DNS in endodontics [12–15], whereas the aim of the present systematic review focusses on the comparison of free hand, static guided endodontics and dynamic navigation system in terms of dentin preservation in endodontic access cavity preparation.

## Methodology

The systematic review was conducted according to the Preferential Reporting Items of Systematic Reviews and Meta- Analysis (PRISMA) 2020 guidelines [16]. The systematic review protocol has been registered in Open science framework (10.17605/OSF.IO/WECPG).

### Research question

The structure research question based on the PICO framework was “Comparative evaluation of dynamic navigation system to freehand and static guided endodontics in endodontic access cavity preparation on the preservation of tooth structure when assessed on permanent human teeth”.

P- permanent human teeth.

I- dynamic navigation system.

C- freehand or static guided endodontics.

O – amount of dentin removal in endodontic access cavity preparation.

### Inclusion and exclusion criteria

#### Inclusion criteria

In vitro studies conducted on permanent human teeth comparing all three i.e. free hand, static and dynamic; or dynamic compared to either of the other two techniques for access cavity preparation were included. Articles published in English and other languages for which English translation was available were included in this review. There was no time period constraints, the article since the time of inception of this technique till June 2023 were included.

#### Exclusion criteria

All in vivo studies and in vitro studies conducted in bovine teeth were excluded. In addition, articles in the form of letters, commentaries or narratives, gray literature, reviews, case reports and surgical guided endodontic procedures were also excluded.

### Search strategy

The search strategy of this systematic review was based on the PRISMA guidelines. A systematic literature search of three databases : PubMed, Scopus and Embase was carried out by two independent authors for all articles published until the end of June 2023. The keywords used for the search strategy and the number of articles retrieved from each database is given in Table 1.

**Table 1:** Keywords used for electronic database search

Keywords	PubMed	Scopus	Embase	Total
(“single rooted teeth” or “anterior teeth” or “lower premolars” or “mandibular premolars” or “resin replica” or “3D printed teeth” or “digitally duplicated teeth” or “virtually replicated teeth” or “virtual teeth model” or “resin model”) AND (“dynamic navigation system” or “dynamic guided endodontics” or “real time guided endodontics” or “RTGE” or “DNS” or “dynamic navigation” or “computer aided technology” or “computer aided navigation” or “image -guided treatment” or “real-time tracking” or “computer-assisted treatment” or “guided endodontic” or “dynamic guide” or “navigation system” or “dynamically navigated”) AND (“Free hand” or conventional or manual or “manual approach” or “conventional approach” or “traditional” or static or “guided endodontics”) AND (“calcified canal” or calcification or “root canal calcification” or “calcified root canal” or obliteration or “obliterated canal” or “root canal” or “dentin preservation” or “access cavity” or “minimal access” or “conservative access” or “dentin removal” or “simulated calcified canals”)	16	144	14	174

### Study selection

The title and abstract of the articles were screened by two independent reviewers (A.L, S.K) to extract the data based on the inclusion and exclusion criteria. The full-text articles were then accessed and reviewed in detail by the reviewers and based on the inclusion criteria, articles that are excluded was documented with the source of evidence. Any disagreements between the reviewers (A.L, S.K, A.S, J.S) were discussed with the reviewers (S.M) and consensus reached.

### Data extraction

The authors then scrutinised and extracted all relevant data from the included articles. The data extracted included various details such as authors, year, interventions, sample size, tooth type, image acquisition methods, DNS system used, number of operators, parameters assessed, evaluation of tooth substance loss in terms of angulation, accuracy and time taken to perform the study.

### Qualitative assessment of the included articles

The methodological quality assessment of each included article was evaluated. A self - designed criteria checklist was formulated based on the checklist by Joanne Briggs Institute [17]. Each full text included article was evaluated based on the different bias domains (randomization, sample size calculation, image acquisition using CBCT, angulation, accuracy and time taken) and based on the level of evidence, the scoring was categorised into low, unclear and high. The overall bias for each included full text article was assessed based on the individual reviewers judgement (A.L, S.K) to the questions based on the different domains. The article was judged as ‘ low’ risk of bias, if all the domain categories were of low risk of bias. On the other hand, if the article had any one of the domains of assessment as ‘high’ it was judged as ‘high’ risk of bias and if it was ‘unclear’ in any one of the domains, it was considered as ‘unclear’. As mentioned earlier, in case of disagreement between the authors consensus was reached by discussion with the third reviewer (S.M).

## Results

**Fig. 1:**
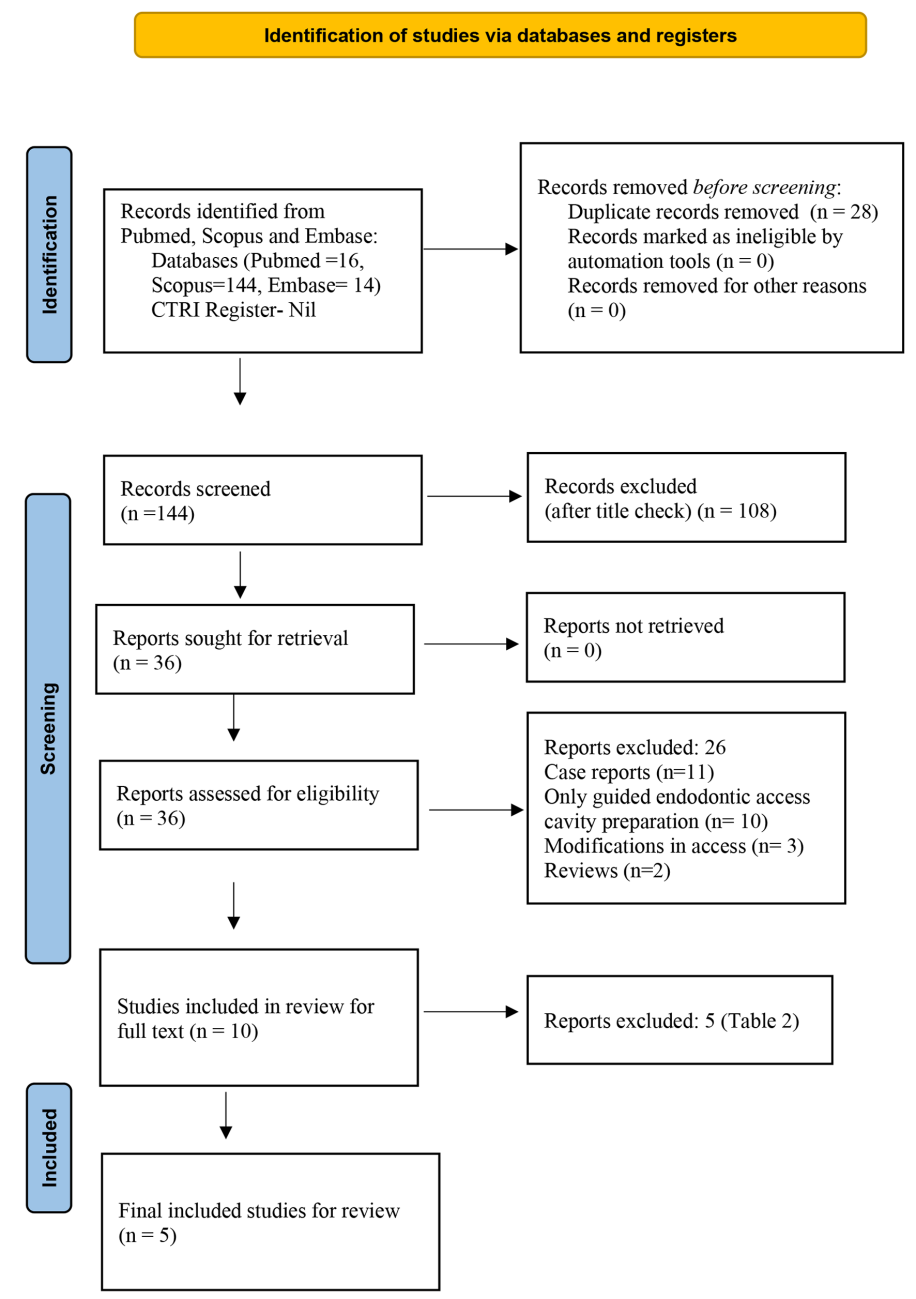
PRISMA 2020 flowchart

The search strategy adopted in this systematic review according to the PRISMA guidelines is depicted in Fig. 1.

### Data extraction

The number of articles retrieved from each database has been represented in Table 1. A total of 174 articles were obtained following the initial search from 3 search engines. After removal of duplicates, a total of 144 articles were obtained. On screening the title, 108 articles were excluded as they did not meet the inclusion criteria. On evaluating the abstract for the 36 articles, only 10 articles were included. The full text of 10 articles were reviewed by the authors, from which 5 articles were excluded because they did not meet the inclusion criteria put forth for this systematic review. The characteristics of the excluded and included studies is tabulated in Tables 2 and 3 respectively.

**Table 2:** Excluded studies with reasons for exclusion

S.No	Author (Year)	Reasons For Exclusion
1.	S D Jain et al. [18] (2020)	Evaluates the 3 Dimensional accuracy of dynamic navigation system in locating calcified canals- no comparator group
2.	B S Chong et al. [19] (2019)	Involves the *invitro* analysis of DNS in locating the canals – no comparator group
3.	Leontiev et al. [20] (2022)	Efficacy of miniaturised navigation system in guided access preparation is assessed- no comparator group
4.	Torres et al. [21] (2021)	Laboratory study evaluating the operator efficiency with the use of DNS
5.	Torres et al. [22] (2023)	*Invitro* study evaluating the operator efficiency with DNS

**Table 3:** Characteristics of the included studies

S.No	Author/ Year	Interventions (Groups)	Sample Size	Tooth Type	Image Acquisition Method	DNS System Used	Number Of Operators	Parameters Assessed	Angulation	Accuracy	Time Taken	Outcome	Main Result
1	Gambarini et al. [23] (2020)	Group 1: Manual (MA) Group 2: DNS	20	Upper molar resin replicas	CBCT	Navident	1	Location of the occlusal starting point Position of the access cavity at the orifice level	DNS = 4.8^0^±1.8^0^ MA = 19.2°± 8.6^0^	DNS = 0.34 ± 0.19 mm MA = 0.88 ± 0.41 mm	DNS = 11.5 ± 2.4 s MA = 12.2 ± 3.2 s	All canals located	DNS exhibited increased precision with significant preservation of tooth structure
2	Álvaro Zubizarreta-Macho et al. [11] (2020)	Group A: Static navigation system (SN); Group B: Dynamic navigation system (DN); Group C: manual (freehand) (MN).	30	Lower central incisors	CBCT (105.0 kV, 8.0MA, 7.20 s, and FOV 15 × 13 mm)	Navident	1	Accuracy of computer aided static vs. dynamic navigation system vs. conventional access preparation	SN = 7.44^0^ ± 1.57^0^ DNS = 3.14^0^ ± 0.86^0^ MN = 4.03^0^ 1.93^0^			All canals located in SN and DNS groups, whereas MN control group showed one root perforation and two missed root canals MN group exhibited one root perforation and two missed root canals	Interms of accuracy DNS > SN > MN
3	Omid Dianat et al. [24] (2020)	Group 1- DNS Group 2- Freehand (FH)	60	Maxillary and mandibular incisors, canines and premolars	CBCT (0.90 mm resolution)	X-Guide system	2	deviations, reduced dentin thickness, and time, end drilling point and procedural errors	DNS = 2.39 ± 0.85º FH = 7.25 ± 4.2º	DNS- BL= 0.19 ± 0.21 mm ; FH- BL = 0.81 ± 0.74 mm DNS- MD= 0.12 ± 0.14 mm; FH- MD = 0.31 ± 0.35 mm	DNS = 227 ± 97 s FH = 405 ± 246 s	DNS- 96.6% (29/30 samples canals located); FH- 83.3% (25/30 samples canals located)	DNS proved to be more accurate and effi cient when compared to than FH
4	Sameer D. Jain [25] (2020)	Group 1- Freehand (FH) Group 2- dynamic navigation system	40	Maxillary and mandibular single-rooted central incisors	CBCT	Navident	1	speed, qualitative precision, and quantitative loss of tooth structure		DNS = 27.2 mm^3^ FH = 40.7 mm^3^	DNS = 136.1 s FH = 424.8 s	DNS showed less tooth substance loss than FH	Based on tooth substance loss, DNS showed significantly less tooth substance loss compared to FH
5	Connert et al. [26] (2021)	Group 1- Real Time guide endodontics (RTGE) Group 2- Conventional (CONV)	72	Central incisor, lateral incisors and canines	CBCT	DENACAM	2	pulp canal calcification using substance loss and procedure time		RTGE group = 10.5 mm^3^ CONV group = 29.7 mm^3^	DNS = 195 s CONV = 193 s	RTGE showed less tooth structure loss when compared to CONV	RTGE showed more accuracy with less tooth substance loss than CONV

### Qualitative assessment of included studies

Of the 5 included studies, one study included all the 3 techniques [18] while the rest compared DNS with either of the other two techniques for access cavity preparation [19–22]. All the studies involved the assessment of image acquisition using CBCT following which the dynamic navigation system was used for access cavity preparation in real time [18–22]. The amount of tooth substance loss were comparatively evaluated where three studies assessed the angulation [18–20], four studies analysed the accuracy and time taken and it was proven that DNS showed increased accuracy, decreased angulation variations and lesser time taken when compared to free hand approach [19–22]. Only one study compared all the three techniques namely DNS, static-guided endodontics and free hand technique and concluded that DNS demonstrated increased accuracy with lesser tooth substance loss in terms of angulation variations when compared to static guided endodontics and free hand technique [18].

### Risk of bias assessment

**Fig. 2:**
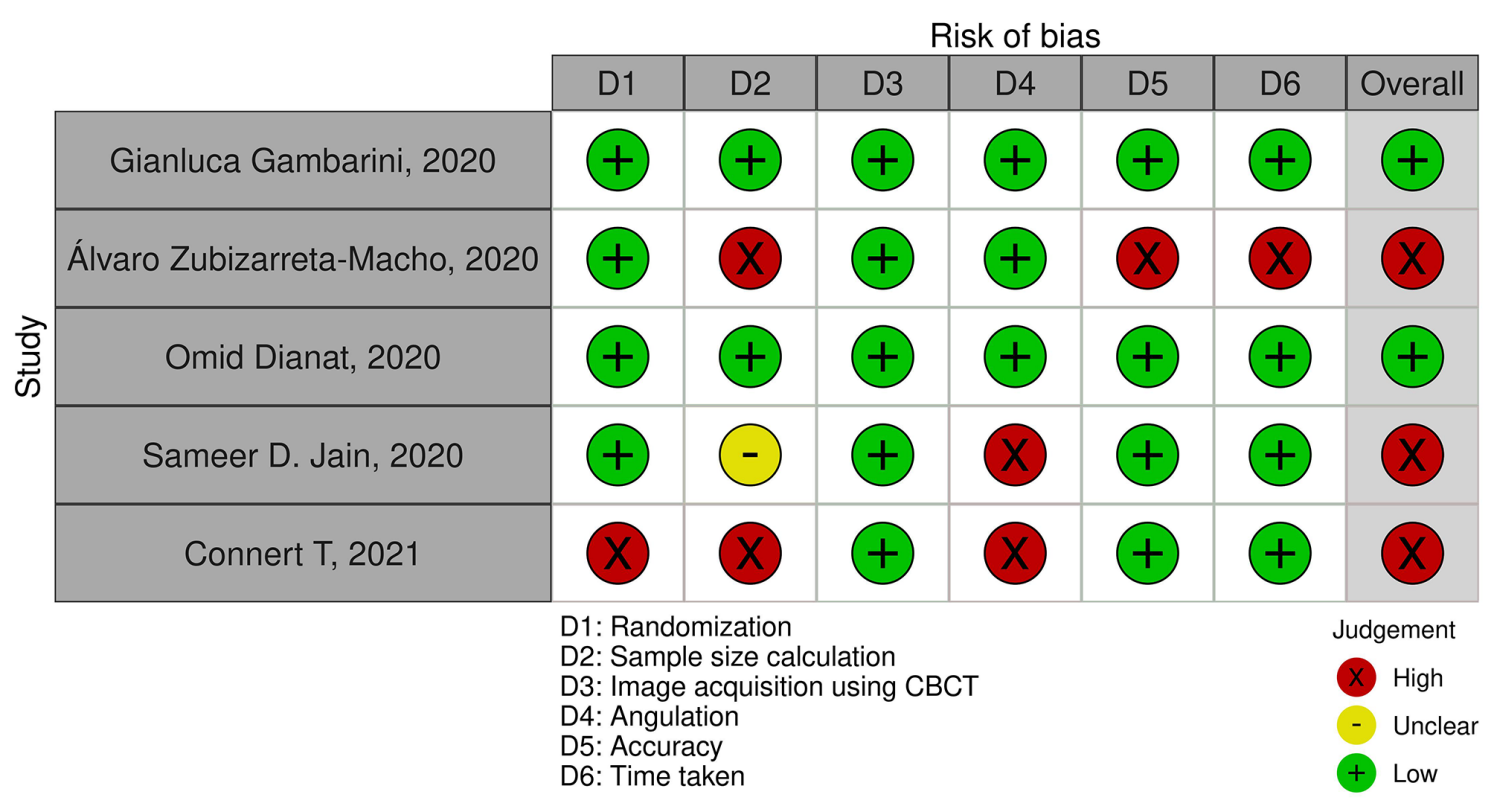
Risk of bias assessment TLC summary plot

**Fig. 3:**
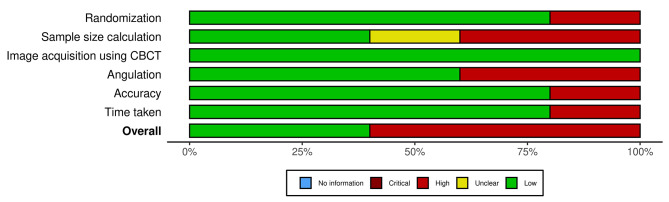
Risk of bias assessment graph

The randomisation, sample size calculation, image acquisition using CBCT, angulation, accuracy and time taken were evaluated in the included full text articles for the risk of bias assessment. The scoring based on the level of evidence for the 5 included articles was tabulated and the summary of the overall risk of bias assessment is represented in Figs. 2 and 3.

Two articles displayed ‘low’ risk of bias [19, 20] and three articles showed ‘high’ risk of bias. The three articles were categorised as ‘high’ risk of bias as the parameters such as randomisation, sample size, angulation, accuracy and time taken have not been evaluated [18, 21, 22]. Macho et al. comparatively evaluated all the three techniques, however based on the outcome assessment for the present systematic review the domains namely sample size calculation, accuracy and time taken were not mentioned [18]. On the other hand, in the study by Sameer D Jain et al., the sample size calculation was unclear and the angulation parameter was not evaluated [21]. The data on the main domains of randomisation, sample size calculation and angulation were not mentioned in the study by Connert et al [22]. Therefore the abovementioned three studies were classified under high risk of bias. As the data in the included articles were heterogenous, it was impossible to conduct a quantitative meta-analysis with the existing data.

## Discussion

Traditional access cavity preparation creates a structural loss weakening the tooth by up to 63% which is weakened by pathologic changes [23]. The amount of enamel and dentin preserved during access cavity preparation favorably influences the biomechanical behavior of the tooth against functional occlusal loading. Higher the volume of coronal wear, higher will be the stress concentration at the cervical region [24]. The loss of peri-cervical dentine due to the excessive removal of dentine during access cavity preparation impacts the internal morphology, deformability and fracture resistance, thereby compromising the prognosis of the tooth following endodontic therapy. The use of magnification decreased the excessive removal of tooth structure during access cavity preparation. However, the suboptimal trajectory below the cementoenamel junction in free hand access cavities detrimentally leads to catastrophic fracture [21, 25, 26].

Advancements in 3-dimensional (3D) printing and surface scanning led to the advent of static guidance system in which the predetermined access drill path using CBCT is transferred to a rigid template [27, 28]. The effective use of CBCT based splint guides diminishes the iatrogenic errors in turn preserving the sound tooth structure. The accurate pre-operative planning by utilising the 3D CBCT images optimises a printed template which is attached to sleeves, guiding the operator to succinctly locate the root canal. Connert et al. reported the prevalence of missed canals which accounted to 8.3% of cases with a mean substance loss of 9.8 mm and the substance loss for conventional access cavity preparation was five times greater than static guided access cavity preparation. Moreover, it is to be noted that the 3D printed root canal was successfully located in 91.7% of guided endodontics cases when compared to 41.7% of conventional endodontic cases [11]. The increased time consumption and cost incurred for intraoral scanning and 3D printing limits its clinical use.

With the era of digitalization, the introduction of Dynamic navigation system has proven to diminish the volumetric tooth substance loss. In DNS, the potential advantage includes the ability to change the direction of the access cavity in real time, improved visibility of dental tissues with preservation of tooth structure and reduces the iatrogenic errors. On comparing DNS with FH access cavity technique, the reduced angular deviation, linear deviation and minimal reduction of dentinal thickness enhanced accuracy and reliability. Hence the present systematic review was undertaken. According to Macho et al., the computer aided static and DNS enhanced the accuracy of endodontic access cavity preparations when compared to the free hand technique [18]. Zehnder et al. reported a mean angle deviation of 1.81^0^, mean coronal deviation of 0.16–0.21 mm and mean apical deviation of 0.17–0.47 mm when the endodontic access cavity preparation was done using a 1.5 mm diameter implant bur. In maxillary teeth, FH endodontic access cavities caused twice the substance loss with twice the suboptimal trajectory when compared with DNS enabled access cavities [29]. Buchgreitz et al. reported that 75% cases of dynamically navigated access and 40% cases of static guided access followed an optimal trajectory. The mishaps of perforation is minimized with the use of DNS which was able to locate root canals in 96.6% of teeth without perforation [3]. Omid Dianat et al. reported that the DNS method improved ease of access cavity preparation with decrease in the time to an average of 4 min (maximum of 7 min) without mishaps. On the other hand, the average time in the FH group for locating canals was 7 to 19 min [20].

Previous systematic reviews, meta-analysis and scoping review by Jonaityte et al., Macho et al. Vasudevan et al. and Martinho et al. have comprehensively evaluated the use of dynamic navigation in guided surgical and nonsurgical endodontics [12–15]. However it is to be noted that the present systematic review focusses on the comparison of the efficacy of free hand, static guided endodontics and dynamic navigation system in endodontic access cavity preparation.

The present systematic review analysis revealed that 10 articles were eligible based on the inclusion and exclusion criteria for further assessment. On full text assessment, the studies by Jain et al., Chong et al., Leontiev et al., Torres et al. and Torres et al. were excluded as the evaluation parameters were not in correlation to the research question put forth in the present systematic review [30–34]. Based on risk of bias assessment analysis, only 2 out of the 7 articles, authored by Gambarini et al. and Omid Dianat et al. demonstrated low risk of bias indicating the insufficient literature evidences promoting the use of DNS over FH and static guided endodontic access cavity preparation systems [19, 20]. On the other hand, the studies by Macho et al., Jain et al. and Connert et al. showed high risk of bias as the parameters such as randomisation, sample size, angulation, accuracy and time taken have not been addressed which are few key pre-requisites for *invitro* access cavity preparation technique analysis. Literature reports reveal that the accuracy, reliability, ease of preparation, real time drilling position and maximal preservation of tooth structure with DNS promotes its opportunity for clinical use [18, 21, 22]. As there are limited *invivo* clinical studies using DNS for access cavity preparation, the present systematic review included evidences from *invitro* studies which is a major limitation. However, an increase in the number of *invitro* studies in the future comparing DNS with FH and static guided endodontics can pave way towards the clinical translation of dynamic navigation system in endodontic access cavity preparation.

## Conclusion

With the limited literature evidences, the present systematic review demonstrates the maximum dentin preservation in terms of angulation, accuracy and time taken during access cavity preparation with the use of dynamic navigation system. However, this needs to be validated with further *invitro* and *invivo* well- designed comparative clinical trials.

## Data Availability

Data is provided within the manuscript.
